# Evaluating the Behavior and Temperament of African Penguins in a Non-Contact Animal Encounter Program

**DOI:** 10.3390/ani9060326

**Published:** 2019-06-06

**Authors:** Sana T. Saiyed, Lydia M. Hopper, Katherine A. Cronin

**Affiliations:** 1 Animal Welfare Science Program, Lincoln Park Zoo, Chicago, IL 60614, USA; sanatsaiyed@gmail.com; 2 Department of Anthropology, University of Notre Dame, Notre Dame, IN 46556, USA; 3 Lester E. Fisher Center for the Study and Conservation of Apes, Lincoln Park Zoo, Chicago, IL 60614, USA; lhopper@lpzoo.org

**Keywords:** *Spheniscus demersus*, welfare, behavior, ambassador animals, temperament

## Abstract

**Simple Summary:**

As animal ambassador programs increase across zoos, it is important to assess the impact they may have on animal welfare. We investigated possible behavioral impacts of a penguin encounter program at the Lincoln Park Zoo in Chicago, Illinois, on a colony of 15 African penguins. We also investigated whether individual characteristics, including temperament, related to penguins’ voluntary participation in the program. We collected 16 weeks of behavioral data, during which 43 Penguin Encounters occurred, which allowed zoo guests to enter part of the penguins’ enclosure. We found no significant differences in colony affiliative or aggressive behavior between days with or without encounter programs, suggesting that offering encounters did not disrupt social behavior in the colony. We also measured penguin temperament on a shy-bold continuum by recording birds’ responses to novel objects and found that temperament, as well as age and sex were predictive of their voluntary participation. We concluded that this program had a neutral or positive impact on penguin welfare, possibly due to aspects of the ambassador program that provided penguins with control over their involvement.

**Abstract:**

Animal ambassador programs are increasingly prevalent in zoos, yet few studies have investigated their impact on animal welfare. We assessed the effects of an ambassador program on the behavior of a colony (*N* = 15) of zoo-housed African penguins (*Spheniscus demersus*) and evaluated whether individual characteristics were predictive of participation. Behavioral data were collected for 16 weeks and included 43 “penguin encounters”, during which zoo visitors entered a designated portion of the penguins’ enclosure. When comparing colony behavior following encounters to behavior during a matched control period lacking an encounter, we found no significant difference between affiliative or aggressive behaviors, suggesting that the encounters did not disrupt interactions in the colony. The same was true when comparing behavior preceding the encounter to a matched control period, indicating that any anticipatory period was similarly non-disruptive. Space use during encounters suggested comfort near visitors. We also measured penguin temperament on the shy-bold continuum by recording the birds’ response to novel objects and found that penguins’ temperament, sex, and age were predictive of participation. We concluded that this program had a neutral or positive impact on penguin welfare and considered the findings in relation to aspects of the ambassador program that provided penguins with control over their involvement.

## 1. Introduction

A primary objective for the Association of Zoos and Aquariums (AZA) is to encourage public action to protect wildlife through engagement with conservation issues, as stated in the AZA Strategic Plan. Some institutions work to accomplish this objective through animal ambassador programs, during which zoo visitors are given the opportunity to learn about animal care, biology, and conservation while in close contact with wildlife [1]. AZA describes animal ambassadors as animals that have been trained by zoo or aquarium staff for public interaction, specifically to support the educational and conservation goals of AZA institutions. These programs may occur with animals in their enclosures, while members of the public are inside or outside the enclosure, or with animals outside of their enclosures. Additionally, the amount of public interaction may vary from close proximity to physical contact between the public and the animals [2].

Ambassador animal programs are a growing form of outreach and engagement at zoos and aquaria, and research evaluating the efficacy of ambassador programs for motivating conservation-related behavior is growing [3]. In a review of studies investigating the effects of zoo-wildlife encounters on visitors, Ballantyne and colleagues [1] found that people were more committed to wildlife conservation after exposure to ambassador programs, presumably mediated by an emotional response to close interaction with the program animals (not necessarily including physical contact). The emotional response generated by ambassador programs may be key to their potential success as a conservation tool, as the emotional connections visitors form can promote learning and knowledge retention and further enable visitors to extend empathy to animals [3,4]. This emotional connection may result in visitors being more open and able to relate to conservation issues facing the animals’ wild counterparts [1,3]. This connection between experience and emotion, which may be fostered through various means, may elevate both the level of educational benefit and the public perception of zoos [5,6,7].

Despite the potential benefits ambassador programs may confer for guest engagement, the impact such programs have on the ambassador animals themselves has not yet been the focus of much empirical research. Although many studies have examined how zoo visitors outside of exhibits affect the behavior and physiology of zoo-housed animals, e.g., [8,9,10,11], few have systematically analyzed the effects of closer encounters between visitors and zoo animals. Baird and colleagues [12] conducted a multi-institutional study using physiological and behavioral measurements of welfare to gauge the effects of ambassador programs on multiple species. The authors investigated whether participating in educational programming or being handled by zoo caretakers was associated with an increase in animals’ levels of fecal glucocorticoid metabolites (FGM) or stress-related behaviors (pacing, rocking, picking or plucking hair/skin/feathers), both of which could be evidence for compromised welfare. In one experiment, the authors compared behavior and FGM levels amongst 59 program, non-program, and off-exhibit armadillos (*Tolypeutes matacus*, *Dasypus novemcinctus*, *Euphractus sexcinctus*, and *Chaetophractus vellerosus*) across 17 AZA-accredited zoos. In a second experiment, the authors studied the effect of handling for programs on behavior and FGM levels of 10 armadillos (*T. matacus* and *C. vellerosus*), six red-tailed hawks (*Buteo jamaicensis*), and 12 African hedgehogs (*Atelerix albiventris*). In both experiments, the authors collected fecal samples every other day and collected daily behavioral data. They found no evidence of increased FGM levels or undesirable behaviors in program animals, but did find a positive relationship between FGM levels and frequency of zoo-caretaker handling, suggesting that an ancillary aspect of ambassador programs (physical handling) may be associated with changes in animals’ physiological indicators of stress.

A second example of an investigation into the relationship between ambassador programs and program animal welfare came from a study by Hartell-DeNardo [13] on the effects of behind-the-scenes tours with zoo-housed Magellanic penguins (*Spheniscus magellanicus*). During these 10- to 20-min tours, visitors could gently pet two penguins. During tours, caretakers would often guide the penguins to walk near the visitors or gently hold the penguins to allow visitors to touch them. The author compared FGM levels and several behaviors, including those indicative of stress or defensiveness, such as nipping and biting, during the weeks the penguins were involved in visitor tours with weeks they were not, for a total of five weeks. The author collected fecal samples opportunistically and collected behavioral data during the tours. Data were collected on five penguins, and the results varied by individual. Two penguins exhibited increased FGM levels on days with tours, while three did not. All five birds exhibited an increase in negative behaviors (nipping, biting), during involuntary situations, such as when guided into a different location by caretakers. This suggests that responses to ambassador programs may vary by individual and that individual control over the level of participation may be a key component to the welfare response.

In the current study, we aimed to evaluate if and how a new educational ambassador program at Lincoln Park Zoo in Chicago, USA, impacted the behavior of captive-born African penguins (*S. demersus*). African penguins are an endangered species [14] and are one of the species prioritized by AZA’s Saving Animals From Extinction (SAFE) program, which seeks to prioritize the protection of threatened and endangered species around the world. Wild populations of African penguins, which are endemic to the coasts of Namibia and South Africa, have decreased by over 90% in the 20th century, and their endangered status is due directly to human activities, such as overfishing and egg exploitation [15]. The direct impact of human activity on their survival makes African penguins an important species for conservation education with zoo visitors.

An evaluation of the welfare of the African penguin colonies used for ambassador programs is essential and timely, as approximately 48 AZA-accredited institutions house African penguins [16] and penguins are a common ambassador species in zoos. Here, we focus on behavioral changes to gain some insight into welfare implications. In order to evaluate the impact of Lincoln Park Zoo’s new ambassador program “penguin encounters” (encounters) on penguin behavior, we collected data during the first year the program was offered and considered whether behavior in the colony differed in relation to whether an encounter program took place. In order to do this, we compared the birds’ behavior from before and after the encounters (pre/post-encounter) to behavioral data collected at the same time of day, but on days without encounters (matched controls). By employing this powerful “matched control” design [17,18,19], we controlled for changes in the birds’ behavior that would be expected throughout the day, due to factors such as fixed feeding times, to isolate changes due to the encounters themselves. We reasoned that behavior prior to the encounter may differ from baseline as penguins anticipated the upcoming encounter, cued by caretaker preparation of the encounter area. We also reasoned that the birds’ behavior following the encounter may be impacted as the colony was reunited. We considered affiliative and aggressive interactions separately in order to have a sense of whether positively- or negatively-valenced social interactions changed as a response to offering encounters. An increase in aggression could signal that the birds’ welfare is being compromised by offering encounters, as aggression is more likely to be associated with negative physical and subjective states such as pain and fear [20,21]. Likewise, an increase in affiliative interactions could signal that the birds’ welfare is being improved by offering encounters, as affiliative interactions in social species may be associated with physiological and affective states associated with good welfare, e.g., [22,23,24].

In addition to considering the birds’ behavior before and after the encounter was conducted, we also summarized the birds’ behavior during encounters. We did not compare this behavior directly to any control since the encounter environment and the behavioral opportunities it provided were unique. Instead, when considering the birds’ behavior during encounters, we focused our question on what aspect of the encounter engaged the penguins. We also considered how penguins utilized the encounter space to draw inferences about their comfort level in proximity to guests.

To build off recent work demonstrating that penguins show individual variation in personality generally [25,26], and in their response to ambassador programs specifically [13], we also considered what individual traits correlated with the birds’ participation in the encounters (if any). Beyond simply considering each bird’s age and sex, however, we also recorded an independent measure of the birds’ boldness (i.e., their willingness to approach a novel object [27,28]). We did this in order to determine if boldness was a good correlate of their participation in the ambassador program. Studies have shown that measures of boldness from novel object tests are consistent across situations [29] and that boldness is positively associated with risk-taking [30]. If boldness, age, or sex correlates with voluntary participation, we will be better positioned to make predictions about which animals may be comfortable in ambassador programs moving forward.

## 2. Materials and Methods

### 2.1. Subjects and Housing

**Figure 1:**
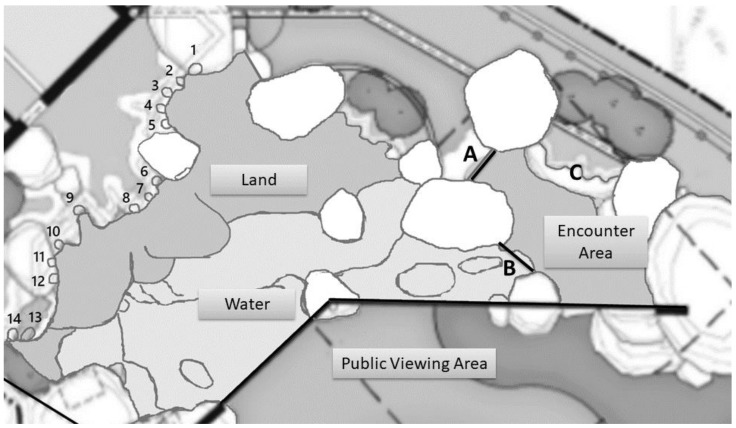
Map of the penguin enclosure. A and B: gates connecting penguin habitat to the encounter area. C: built-in bench for guests. Numbers 1–14 indicate the position of the 14 nest boxes to which the colony had continuous access.

The African penguin colony at Lincoln Park Zoo consisted of 15 penguins: 10 males and 5 females. The average age of the penguins was 6.5 years, ranging from 2–13 years. The colony arrived at Lincoln Park Zoo in 2016, with the opening of a new exhibit: the Robert and Mayari Pritzker Penguin Cove. The penguins came from multiple institutions, and none had been ambassador animals previously. Penguins were observed in the outdoor exhibit, which was 331.23 m^2^ and included a 77,600-L pool and 14 nest boxes (71 cm L × 53 cm W × 56 cm H) (Figure 1). Each day, the animal caretakers provided different forms of enrichment in the exhibit, such as plastic pool toys, pebbles, and grass, or a water sprinkler. The penguin colony received two hand-feedings per day at 09:00 and 14:00.

### 2.2. Penguin Encounters

Each encounter began with an informational session conducted in front of the penguin exhibit viewing window, during which staff from the zoo’s Learning Department discussed African penguin habitat, behavior, anthropogenic threats, and the importance of African penguin conservation for about 20 min. The visitors were then provided with instructions on how to behave during the encounter; specifically, they were instructed to put on the provided rubber boots and told that they should sit quietly on the bench that ran along one side of the encounter space with their hands in their lap and to not touch the penguins. Visitors were also told that they could not use their phones or take their own photos during the encounter. After receiving this information, visitors entered a partitioned portion of the penguins’ exhibit, the “encounter area” (Figure 1). Outside of encounter times, penguins had full access to this area. Each encounter involved up to 10 visitors over the age of six years old (mean number of visitors per encounter = 5, range = 1–10, average age = 37.8, range = 6–85). Once visitors were seated on the bench inside the encounter area, a caretaker opened one or two gates to allow 1–3 penguins entry into the encounter area. Often, the caretakers used enrichment items, such as a feather, to increase the penguins’ interest in entering. However, caretakers never walked behind penguins to encourage movement into the encounter area. Once 1–3 penguins had entered, the caretaker closed the gate(s) and kept both gates closed until the encounter ended or any penguin signaled they wanted to leave the area (see next paragraph). During the encounter, caretakers engaged the penguins with enrichment items, such as toys or laser pointers, while visitors observed the penguins. From time to time, caretakers (but not visitors) would pet or touch the penguins upon their approach. Caretakers also relayed information about captive penguin care and individual penguin behavior to the visitors.

To maintain the voluntary nature of the program, penguins were permitted to leave at any time during the encounter by walking toward either gate (Figure 1) and remaining there for a few seconds. At this time, a caretaker would open the door to allow the penguin to return to the colony. As such, participation in the encounter was voluntary for the penguins, and the composition of penguins participating in the encounter was not predetermined. The encounters were offered up to twice daily at 10:00 and 15:00 (each lasted about 1 h, including the 30-min informational session prior to entering the encounter area), April–October 2017, but were cancelled if visitors did not register. As such, encounters were not a daily occurrence.

### 2.3. Behavioral Data Collection

We used two observation protocols: one to record behavior when encounters were not occurring (i.e., for the pre-/post-observations and their corresponding matched control periods) and one to record the birds’ behavior while encounters were occurring. All data were collected by a single observer (the first author) standing in the public viewing area on a handheld tablet using the behavioral recording app, ZooMonitor [31,32].

**Figure 2:**
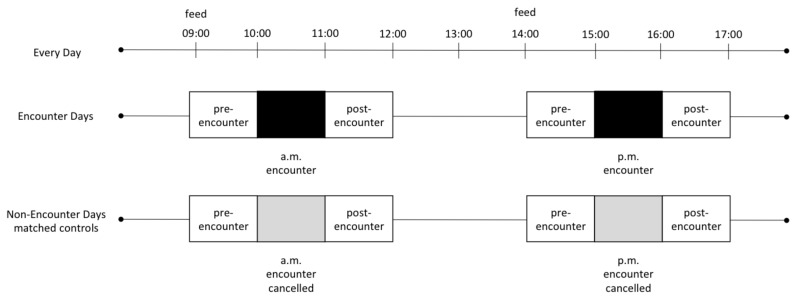
Study design, represented as a timeline for behavioral data collection. Mornings or afternoons when encounters occurred are represented by black boxes; mornings or afternoons when encounters were cancelled are represented by grey boxes. Pre-encounter and matched control pre-encounter data were collected from 09:00–10:00 and/or 14:00–15:00. Post-encounter and matched-control post-encounter data were collected from 11:00–12:00 and/or 16:00–17:00.

Outside of encounter times, observations of the colony’s behavior consisted of 10-min sessions. During each observation session, we recorded behavioral data on all visible penguins simultaneously via an all-occurrence basis [33], following a standardized ethogram (Table 1) that was designed to prioritize behaviors of interest to the evaluation of welfare and behaviors directed toward humans. We recorded the visibility of all penguins at two-minute intervals using the map feature of the ZooMonitor app (visible individuals were plotted; non-visible animals were not). We used this point sampling data to estimate the number of minutes each individual was visible and subsequently used them to standardize behavioral rates by visible time per individual. Data were collected on weekdays between July and October 2017, between the hours of 09:00 and 12:00 and 14:00 and 17:00 (Figure 2). In total, 74 h of behavioral observations were conducted for this study on 57 different observation days. Thirty-three of the observation days included either an a.m. or a p.m. encounter (but not both), while 10 included both an a.m. and a p.m. encounter, and on 14 days, there were no encounters.

For the behavioral observations conducted during encounters, we conducted sessions matched in length to each encounter (up to 30 min). We recorded behavioral data of penguins in the encounter area on a continuous basis, following an ethogram designed to provide information about the degree to which penguins directed their attention toward caretakers, enrichment, visitors, or themselves while in this close contact environment (Table 2). This continuous sampling protocol was facilitated by the simplified ethogram, the reduced area in which the penguins were observed (i.e., the encounter space, not the entire exhibit), and the small number of penguins that participated in the encounters. We also used the ZooMonitor app to record the location of penguins within the encounter space with point sampling at one-minute intervals to gain a better understanding of whether penguins showed a preference or attraction toward the area where visitors were seated. Observations were conducted from 7 July–21 October 2017, from 10:30–11:00 and/or from 15:00–15:30. Twenty hours of behavioral data were collected on a total of 43 days.

### 2.4. Temperament Tests

We conducted a novel object temperament test in order to assess whether boldness predicted penguin participation in the encounter program [30,34]. In order to assess this, we conducted two test trials and three control trials. During test trials, a familiar caretaker sat on a low stool inside the exhibit facing forward, holding a novel object in his/her lap, and looking straight ahead. The location where the caretaker sat was the same as where he/she typically sat during the penguins’ twice-daily feeding sessions. Control trials were identical, except that they lacked the novel object and the caretaker held their hands in their lap. The novel object we presented in the first novel object test was a medium-sized, brown cardboard packaging box (approximately 25 cm L × 25 cm W × 12 cm H), and in the second test, we used an inflatable, grey, plastic shark toy (46 cm L, Play Visions, Woodinville, WA, USA). All trials lasted 5 min, starting when the caretaker was seated on the stool. We ran each trial on a separate day, with all trials run in one month midway through the encounter season (August).

To analyze the novel object temperament tests, we coded, from video, each penguin’s latency to first approach within 1 m of the caretaker during both the test (novel object) and control sessions. Penguins that never approached the 1-m zone were assigned the maximum latency possible, 300 s, for analysis. We used Behavioral Observation Research Interactive Software (BORIS) to code the sessions [35]. We measured test-retest reliability by calculating that latencies to enter the one-meter zone were reasonably correlated between the two test trials (*N* = 15, R^2^ = 0.217, *p* = 0.08). We interpreted this positive relationship that trended toward significance to be indicative that the two novel objects were eliciting similar responses from the penguins, but acknowledged that the relationship failed to reach significance at the traditional *p* < 0.05 level, which we consider in our interpretation of the results below. For each penguin, we then calculated the difference in mean latency to approach the caretaker during control and test trials for each penguin (i.e., a latency difference score). If individuals were faster to approach the 1-m zone during test trials relative to control trials, they would have a value greater than zero, and larger values would be more “bold” on the shy-bold spectrum [30,34]. To determine whether temperament predicted the penguins’ likelihood of voluntary participation in the encounters, we measured whether there was a correlation between the latency difference score of each penguin and the number of encounters in which a penguin participated.

### 2.5. Data Analysis

We categorized the behavioral data collected outside of encounter times based on the time period during which the observations took place to address our questions of interest (Figure 2). Specifically, we categorized data collected within one hour prior to the start of an encounter as pre-encounter observations and data collected within one hour following the end of an encounter as post-encounter observations. Likewise, we categorized data collected at the same times of day, but that did not precede or follow an encounter (i.e., because there were no registered participants and the encounter was cancelled) as matched control pre-encounter and matched control post-encounter data, respectively, following de Waal and Yoshihara [17]. This matched control approach allowed us to largely isolate the effects of the encounters on behavior and adjust for changes in behavior that may be due to time of day. From these datasets, we aimed to extract both the birds’ affiliative and aggressive interactions. We considered all affiliative behaviors collectively (allopreening, courtship, and mounting) and all aggressive behaviors collectively (biting and chasing) (see Table 1 for the definitions). For analyses, we calculated the rates of behaviors (events/10 min) for all affiliative behaviors combined and all aggressive behaviors combined. Importantly, we calculated these behavioral rates based on visible time for each individual. We compared pre-encounter data with matched control pre-encounter data and post-encounter data with matched control post-encounter data. To do so, and given the non-normal distribution of our behavioral data, we used within-subject Wilcoxon signed-rank tests to test for differences in behavioral rates to determine whether hosting encounters impacted penguin behavior. Specifically, we analyzed whether rates of aggressive behaviors, affiliative behaviors, or person-directed behaviors differed between times with and without encounters.

We summarized the behavioral data we obtained during encounters with descriptive statistics based on the duration of time each bird engaged in each behavior as a proportion of time visible. Additionally, we used ZooMonitor to visualize the birds’ individual space use densities within the encounter space during the encounters.

To test the relationship between the birds’ encounter participation and individual characteristics, we calculated nonparametric Spearman rank order correlations for continuous factors (age and temperament) and Mann–Whitney U-tests for categorical factors (sex). All analyses were conducted using R (Version 3.3.3) [36].

This study was approved by the Lincoln Park Zoo Research Committee, the governing body for all animal research at the institution (No. 2017-005).

## 3. Results

### 3.1. Behavior before and after Encounters

We analyzed affiliative behavior and aggressive behaviors to understand if the ambassador program impacted colony interactions. The average individual percent time visible outside of encounter times was 60.9% (±SEM 6.7%, range 26.1–100%). Overall, rates of affiliation and aggression were low across all time periods. The means, standard errors, and results of the Wilcoxon signed-rank test are provided in Table 3. Neither affiliation, nor aggression rates were significantly different depending on whether encounters took place (all *p* > 0.05). Similarly, rates of human-directed behaviors also did not differ based on whether encounters were offered. Human-directed behaviors were predominantly in the form of attention paid toward zoo visitors through the viewing glass. Thus, offering encounters did not appear to influence colony affiliation, aggression, or behavior directed toward zoo visitors.

### 3.2. Behavior During Encounters

At least one penguin participated in every encounter for which guests were registered. While six of the 15 penguins never participated in any encounters, nine penguins participated in at least one encounter during the four-month study period. The three penguins who participated in more encounters than any others were all two-year-old males (participation count for Aje = 13, Erik = 36, Phil = 43). At least one penguin voluntarily left the encounter early in 15 of 53 encounters, and there were 41 individual penguin exits in total (average 0.28 exits per encounter).

While in the encounter, the penguins were visible to the observer 100% of the time due to the layout of the encounter space. For the nine penguins who participated in the encounter at least once, the average (±SEM) percent of time they engaged with enrichment was 23.5% (±6.6%), in affiliative or aggressive behaviors with conspecifics was 0.2% (±0.1%), with keepers was 2.6% (±1.3%), with visitors was 0.5% (±0.2%), and with self (preening) was 5.9% (±3.2%). They spent 67.2% (±8.5%) of time not obviously engaged with any conspecific, enrichment object, or person (i.e., recorded as “other”). Although we did not break this category of behavior down in our ethogram, we informally and retrospectively report that, when recorded as “other”, the penguins were typically standing still or, less frequently, locomoting around the encounter space.

**Figure 3:**
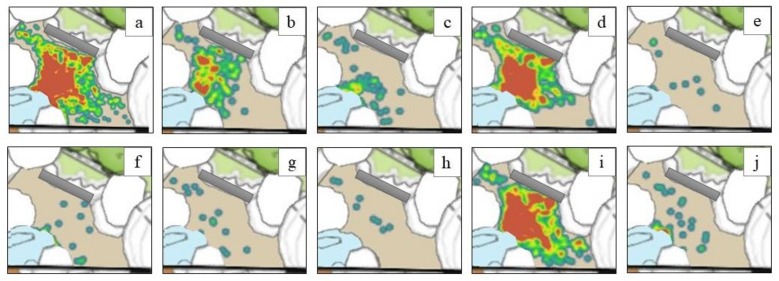
Space use of penguins during the encounters. These space use densities indicate the frequency with which penguins were observed at specific locations inside the encounter area, coded at 2-min intervals during encounters. A single use of a specific location is indicated in blue, and the color changes from blue to green to yellow to red as more use is coded in the same location. The grey rectangle represents the visitor bench. Data shown only for penguins who were observed in at least one encounter. (**a**) All penguin use combined, (**b**) Aje, (**c**) Dudley, (**d**) Erik, (**e**) Liam, (**f**) Mandela, (**g**) Maria, (**h**) Maynard, (**i**) Phil, and (**j**) Preston.

The locations of penguins while in the encounter space can provide insight to their level of comfort; therefore, heat maps of penguin space use during encounters are shown in Figure 3. Figure 3a shows the combined data for all penguins participating in the encounters, and Figure 3b–j shows the data for each individual penguin that participated in the encounter program. The bench upon which visitors sat while penguins were present in the encounter space is indicated in each panel by a grey rectangle. Visual inspection of the space use densities suggests that the participating penguins spent the majority of their time in the center of the encounter area, which was where the keepers often stood and where most of the enrichment was offered. It is also apparent from the space use densities that penguins spent less time near the visitor glass at the front of the habitat. Finally, the space use densities showed that penguins did not avoid the seating area where encounter participants were located, suggesting they were comfortable in proximity to the participants.

### 3.3. Individual Characteristics

**Figure 4:**
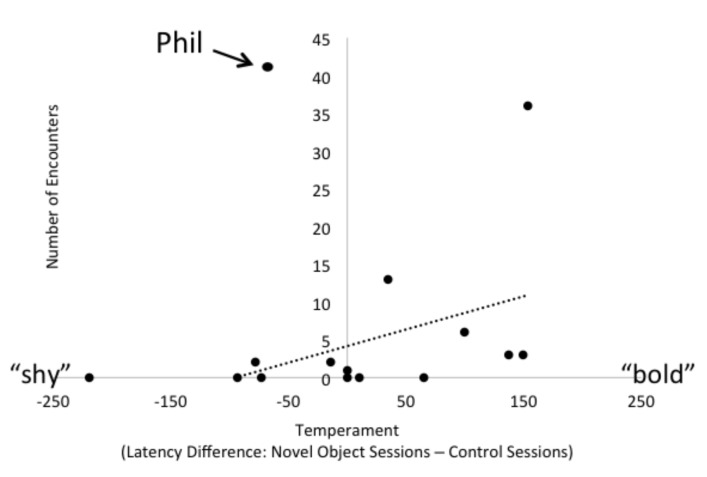
The relationship between temperament and participation in encounters. Penguins who scored higher on the shy-bold continuum tended to participate voluntarily in more encounters. The trend line shown here does not include Phil, a potential outlier.

There was marked variation across the 15 penguins’ temperaments, as measured by our novel object tests of shy-boldness (Figure 4). When assessing the relationship between temperament and voluntary participation in the encounters, we considered the data with and without one potential outlier (Phil), who participated in more encounters than any other penguin (81% of the encounters, more than two standard deviations above the mean). For the remaining 14 penguins, results indicated that their boldness rating, as measured by latency-difference scores, was positively correlated with the number of encounter sessions in which they chose to participate (*N* = 14, r_s_ = 0.436, *p* = 0.010, Figure 4). If the potential outlier is not excluded, the correlation is not significant (*N* = 15, r_s_ = 0.179, *p* = 0.117). See Figure 4 for an indication of Phil’s data in relation to the rest of the colony. We also considered whether age or sex predicted participation in encounters, again with and without Phil. We found that there was a possible trend for younger penguins to participate more often than older penguins, which was weakened when excluding Phil (*N* = 15, Spearman r_s_ = 0.222, *p* = 0.076; excluding Phil, *N* = 14, Spearman r_s_ = 0.168, *p* = 0.145). We also found that males participated more than females, and this relationship was significant when Phil was excluded, but reduced to a trend with Phil included (Mann–Whitney *N* = 10 males, 5 females, U = 7.5, *p* = 0.038; excluding Phil, *N* = 9 males, 5 females, U = 7.5, *p* = 0.054).

## 4. Discussion

We analyzed 94 h of behavioral observations to determine the potential influence of an animal ambassador program on a zoo-housed colony of African penguins. From our observations, we found no evidence that offering the program impacted behavioral interactions in the colony, with no significant difference between rates of affiliative or aggressive behaviors in relation to whether an encounter program took place on a given day. Rates of social interactions were low, but typical for activity budgets previously reported for *Spheniscus* species [37]. Caretakers fed the colony toward the beginning of the pre-encounter period during both encounter and matched control conditions (Figure 2); however, the time-matched design of this study controlled for potential influences feeding had on colony behavior. Rates of behavior directed toward humans, and primarily zoo visitors, were higher than rates of behavior directed toward conspecifics, but did not differ based on whether encounters were offered. Affiliative and aggressive behaviors were also uncommon during the encounter sessions, and the penguins’ use of space suggests comfort in proximity to the guests participating in the encounter. We found that individual penguins differed in their voluntary participation in the ambassador program, and that this difference was predicted to some degree by individual temperament, age, and sex, with a tendency for bolder, young males to participate more often. The results suggest that the colony of 15 African penguins at Lincoln Park Zoo was not exhibiting signs of compromised welfare as measured through changes in behavior, and that temperament tests may be one useful way to predict which penguins are more comfortable in participating in ambassador programs.

Our results differed from the one previous study that investigated penguin behavioral responses to an ambassador program in which Magellanic penguins showed an increase in negative or aggressive behaviors during guest encounter programs [13]. This difference may be due to broad species differences in response, both physiologically and behaviorally, to human presence, e.g., [38,39,40,41]. However, given that both Magellanic and African penguins have been shown to desensitize similarly to human presence in the wild [41,42], additional factors may contribute to the different responses to ambassador programs reported here and by Hartell-DeNardo [13].

We speculate that the African penguins in our study may have exhibited comfort in the encounter space and minimal changes in behavior due to the voluntary nature of the program. Penguins were encouraged to participate by showing enrichment items; however, they were never herded or carried into the encounter area, and they were given the option to leave midsession (which they did often). The responsiveness of the caretakers to the penguins’ choices to leave was likely integral to the birds’ willingness to participate voluntarily. In a study investigating undesirable behaviors in the presence of visitors at a petting zoo, Anderson and colleagues [43] found that animals exhibited fewer undesirable behaviors when allowed to retreat from visitors at will. Additionally, zoo visitors were not permitted to touch or interact with the encounter penguins at any point, which may have increased penguin comfort and allowed them to maintain control over the situation. Related, studies focused on zoo-housed little penguins have revealed that little penguins (*Eudyptula minor*) increased behaviors indicative of visitor avoidance when exhibits were open to the public rather than closed [44], and the same species showed a reduction in fear-related behaviors when visitors were kept at a greater viewing distance [45] (this Issue). Another aspect of the program that was distinct from previous studies was that the program took place in a familiar, dedicated area of the penguin habitat, requiring no animal transport for participation. This is important given the wealth of data that showed the positive benefits of providing captive animals with increased choice and control reviewed in [46]. At this point, we can simply say that these were the conditions under which we measured penguin behavior; moving forward, additional work would be necessary to determine what combination of these program components had the biggest impact.

Some studies on ambassador programs with other species have reported negative behavioral responses to handling, including nipping and biting [3,43]. In this study, we did not differentiate between positive and negative behaviors directed toward keepers during the encounter, but can report anecdotally that they were generally positive and often took the form of leaning against keepers while keepers gently petted them; nipping and biting at keepers were rare events. Specifying the type of interaction was not feasible in this study given the continuous recording of behavior, but should be a priority in future work to allow more quantitative, objective conclusions to be drawn about the nature of the keeper–animal interactions and the relationship that interactional style may have on welfare, e.g., [47]. It would also be important for future work to include a larger sample size to detect smaller, but potentially meaningful differences in behavior between conditions; the current study was sufficiently powered to detect large effect sizes, but more subtle effects could have been missed. It is also interesting to note that penguins in this colony showed frequent interest in zoo visitors outside of encounter times, captured through our measure of human-directed behaviors. Whether this is a trait generalizable to other zoo-housed African Penguins or specific to our colony and whether this trait relates to the degree to which our colony responded positively to the encounter experience remain to be determined with additional research encompassing colonies at other institutions.

Not only do our results stress the importance of monitoring animals’ behavior before, during, and after encounters with zoo visitors, they also highlight the inter-individual variation that animals express in their willingness to engage in animal ambassador programs. Of the 15 penguins in the colony, only nine ever chose to participate in an encounter session during this first season. Furthermore, three of those nine penguins were much more regular participants, with one individual, Phil, joining 43 of the 53 encounters we observed. We attempted to measure the penguins’ inter-individual variation in temperament via novel object tests and found that boldness may have been positively associated with their likelihood of entering the encounter area. This pattern is consistent with previous work that has demonstrated individual differences among penguins [13,25,26] and the relationship between animals’ rating on the shy-bold continuum and other behavioral tendencies [48,49,50].

However, there are reasons to interpret the link between temperament and participation with caution. First, the most frequent participant was rated as a shyer individual, in contrast to the general pattern we found, indicating that shyer penguins participated less often than bolder penguins. We also found that younger penguins, and male penguins, tended to participate more often. Again, we report these patterns as tentative given the influence of a single data point (Phil) on these patterns, which likely reflects limited power related to our small sample size. In our colony specifically, there was a male-biased sex ratio with twice as many males as females, so males may have been more prone to participation because there were not enough potential pair bond partners available in the colony. In the future, longitudinal research with a larger sample size, perhaps facilitated by multi-institutional collaboration, will have to determine what combination of these dimensions (temperament, sex, age, and possibly pair bond status) is best for predicting individual differences in participation. Regardless of the relative contribution of these various dimensions, it is evident that penguins within a single colony will vary in rates of voluntary participation and that programs should be responsive to these individual preferences to ensure good welfare.

## 5. Conclusions

In conclusion, our results indicate that the encounter program did not generate any measurable, adverse behavioral effects. We strongly recommend that these results be considered as only reflective of the conditions under which our program was run, that is a non-contact, non-transport program that provided penguins with the choice of whether or not to participate. Our study demonstrate the usefulness of behavioral data and space use as indicators of animal welfare, as well as one seemingly successful implementation of ambassador programming. The results also demonstrate that simple temperament tests may prove useful in predicting which penguins will be comfortable participating in ambassador programs, although more work is needed in this arena. We acknowledge that animal welfare is a holistic concept, and for a more complete picture, one could consider additional behaviors, physiological data such as endocrine or heart rate measures, as well as indications of mental state or emotional well-being. Moving forward, research programs that integrate additional measures into experimental approaches with different species and varying levels of program intensity will provide a more complete impression of the welfare of animals in ambassador programs.

## Figures and Tables

**Table 1:** Ethogram for data collection while encounters were not occurring.

Behavioral Category	Behavior	Description
Affiliative	Allopreening	Animal is licking or manipulating the skin or fur of another animal in an affiliative context.
	Mounting	Animal is mounting or attempting to mount another individual (intromission may or may not be achieved).
	Courtship	Animal approaches another individual with eyes partially closed, head wag, feathers erect.
Aggressive	Contact Aggression	Animal physically attacks another individual, including biting, batting, or kicking.
	Non-Contact Aggression	Animal performs aggressive behaviors without making physical contact, such as threat displays, lunges, etc.
Person-directed	Keeper Directed	Animal directs any behavior toward the keeper: rubbing, biting, nipping, etc. Interaction could be positive or negative.
	Visitor Directed	Animal directs any behavior toward a zoo visitor in the public area through the exhibit window: following fingers, biting window, etc. Interaction could be positive or negative.

**Table 2:** Ethogram for data collection on penguins participating in encounters.

Continuous Behavioral Category	Description
Engaged with Keeper	Penguin is engaged with keeper: rubbing, biting, nipping, etc. Interaction could be positive or negative.
Engaged with Visitor	Penguin is engaged with visitor: biting or nipping at visitor’s feet/legs. Interaction could be positive or negative.
Engaged with Enrichment	Penguin is engaged with the enrichment involved with the encounter: toys, laser pointer, bubbles, etc.
Engaged with Conspecifics-Aggressive	Penguin is engaged in apparent aggressive behavior (nipping, biting, etc.) toward another penguin in the encounter.
Engaged with Conspecifics-Affiliative	Penguin is engaged in apparent affiliative behavior (courting, mounting, allopreening, playing) toward another penguin in the encounter.
Preening	Penguin is self-preening.
Other	Penguin is performing a behavior that does not fall into a behavioral category listed above (standing, sleeping, etc.)

**Table 3:** Mean behavior rates (events/10 min) for aggression and affiliation during pre-encounter, post-encounter, and matched control conditions and sample sizes, Wilcoxon signed-rank T- and *p*-values.

Behavior	Pre-Encounter Behavior Rate (Mean Events per 10 min ± SEM)			Post-Encounter Behavior Rate (Mean Events per 10 min ± SEM)		
	Encounter Offered	Matched Control	Wilcoxon (all *N* = 15)	Encounter Offered	Matched Control	Wilcoxon (all *N* = 15)
Aggression	0.08 (±0.03)	0.06 (±0.02)	T = 4, *p* = 0.79	0.13 (±0.03)	0.11 (±0.20)	T = 27.5, *p* = 0.58
Affiliation	0.02 (±0.01)	0.01 (±0.01)	T = 3, *p* = 0.35	0.01 (±0.01)	0.00 (±0.00)	T = 3, *p* = 0.35
Person-directed behavior	0.56 (±0.17)	0.53 (±0.26)	T = 27.5, *p* = 0.59	1.12 (±0.37)	0.99 (±0.42)	T = 35, *p* = 0.48
